# Nutrition literacy assessment in older adults: a scoping review of measurement tools

**DOI:** 10.3389/fnut.2025.1608558

**Published:** 2025-09-09

**Authors:** Yuxiao Ren, Yahan Liu, Kai Liu, Runhao Jin

**Affiliations:** School of Nursing, Yanbian University, Yanji, Jilin, China

**Keywords:** older people, nutrition literacy, nutritional status, assessment tools, scope review

## Abstract

**Introduction:**

Nutritional diseases, common among the elderly, are profoundly affected by nutrition literacy. Nutrition literacy influences dietary habits and behaviors; therefore, developing nutrition literacy is essential for alleviating various illnesses and enhancing quality of life. This study sought to perform a scoping review to find and compare techniques for assessing nutrition literacy in older persons and identify any gaps in this area of scholarship.

**Methods:**

A comprehensive search of seven internet databases, including PubMed, Web of Science, the Cochrane Library, Wanfang, China Biology Medicine disc (CBM), China National Knowledge Infrastructure (CNKI) and VIP Database for Chinese Technical Periodicals (VIP), resulting in 1,995 articles. In addition, additional relevant studies were retrieved from the identified articles’ references. Ultimately, 14 publications were included, encompassing a total of 12 nutrition literacy evaluation instruments.

**Results:**

The Simplified Nutrition Appetite Questionnaire (SNAQ), and Nutrition Literacy Assessment Tool (NLAT) were identified as unidimensional instruments, while the other tools had multidimensional attributes. Of the 11 instruments assessed for reliability, the Portuguese version of the Mini-Nutritional Assessment (MNA Portuguese Version) was validated exclusively for validity, lacking a reliability evaluation. Moreover, the NLAT underwent just reliability testing, with no validity assessment performed. We observed that current assessment tools have shortcomings in terms of cultural appropriateness and need further validation. Most current techniques primarily focus on fundamental nutritional knowledge, overall nutritional status, and dietary choices, providing wide-ranging applicability. However, the existing assessment tools exhibit limitations regarding their applicability, indicator systems, and assessment content for elderly patients with chronic diseases, inadequately addressing the specific issues associated with these conditions.

**Conclusion:**

To address clinical nutritional requirements, it is essential to enhance the cross-cultural validation of tools via multidisciplinary translation teams and adherence to international guidelines, thereby improving their applicability across diverse populations. There is an immediate necessity to develop adaptable and customizable assessment instruments for certain diseases in the senior population. These tools should incorporate indicators of nutritional knowledge relevant to particular diseases, along with dynamic monitoring indicators of nutritional status, to enhance personalized nutritional management support for elderly. The integration of artificial intelligence technology into these tools can enhance their optimization, leading to improved accuracy and effectiveness in clinical nutritional practices for elderly care.

## 1 Introduction

The global aging population has emerged as a significant challenge of shared concern (1). The aging trend is widespread, irreversible, and expected to continue for the next two to three decades. The World Health Organization (WHO) predicts that the population aged 80 and beyond will reach 426 million between 2020 and 2050 (2). Against the backdrop of an increasingly aging population, nutrition and health management play an increasingly important role in elderly care work. Related studies have shown that insufficient food intake, lifestyle habits, and other factors are closely related to the occurrence of chronic diseases, with over 70% of chronic diseases being caused by these factors (3–5). Consequently, it is essential to understand nutritional concepts and cultivate healthy eating habits to successfully avoid and manage chronic diseases in older adults (6).

Nutrition literacy, a multi-faceted concept within nutrition and health, refers to an individual’s capacity to gather and assess fundamental nutritional information and to make informed scientific decisions based on established nutritional knowledge. Its objective is to mitigate the risk of diseases such as diabetes and cardiovascular conditions while enhancing chronic disease management to promote both physical and mental wellbeing (7). Multiple studies have established that improving an individual’s nutrition literacy is crucial for treating diet-related chronic diseases, including obesity, diabetes, and cardiovascular diseases, and can directly influence their management efficacy (8–10). Nutrition literacy is a crucial prerequisite for establishing healthy dietary behaviors, significantly influencing individual development and directly correlating with enhancements in dietary practices as nutritional knowledge increases (11, 12). Nutritious dietary practices promote healthy growth, whereas detrimental eating behaviors, including inadequate consumption of fruits and vegetables and excessive intake of high-sugar, high-fat, and high-calorie foods, can disrupt food choices and consumption, elevate the risk of malnutrition, obesity, and deficiencies in essential vitamins or minerals, and potentially result in eating disorders (13). Particularly among the elderly, a lack of nutritional literacy hampers their ability to tailor diets according to their health conditions, rendering them susceptible to misleading or erroneous nutritional information. This can culminate in an inappropriate dietary framework, heightening the risk of deficiencies in energy, protein, vitamins, or minerals, ultimately leading to malnutrition. From the standpoint of pathology and nutrition, older adults with insufficient nutrition literacy are more vulnerable to deficiencies in essential nutrients such as vitamin D and calcium, leading to a significantly increased risk and prevalence of malnutrition, which is closely linked to dietary practices that do not meet nutritional needs (14–16). These variables can exacerbate the deterioration of physical and mental functions in the aged, significantly affecting disease prognosis. Nutritional deficiency in hospitalized elderly individuals can result in increased risks of infection and mortality, prolonged hospital stays, reduced efficacy of pharmacological treatments, elevated readmission rates, and rising medical costs, thereby placing a considerable burden on family members (17). Furthermore, epidemiological and comprehensive community studies have shown that poor dietary habits can substantially exacerbate the prevalence of chronic non-communicable diseases, such as type 2 diabetes (T2DM), hypertension, cardiovascular diseases (CVD), cancer, neurodegenerative disorders, arthritis, chronic kidney disease (CKD), and chronic obstructive pulmonary disease (COPD) (18).

Given the correlation between nutrition, aging, and diseases, it is crucial to evaluate the nutrition literacy of the elderly. As countries such as Japan and the United States have made significant trides in identifying influencing factors and developing assessment tools (19–21). Nonetheless, research on nutrition literacy among older people in some countries is in its infancy. Current instruments for evaluating the nutrition literacy of the elderly are many and extensively utilized, encompassing various contexts such as communities, hospitals, and nursing facilities. The evaluative perspective can be classified as either subjective or objective. The subjective perspective includes factors such as the dietary health knowledge, health beliefs, and attitudes of older adults. The objective approach depends on empirical data to evaluate the anthropometric indicators, biochemical markers, health state, and lifestyle habits of elderly individuals. The integration of objective data and subjective assessments facilitates a thorough assessment of nutritional knowledge in older persons. However, the prevailing nutrition literacy assessment tools are characterized by a high degree of generalization, resulting in a lack of disease specificity, as evidenced by their inability to comprehensively and accurately reflect the nutritional status of elderly diabetic patients. Additionally, there is a paucity of systematic analysis and summarization of the cultural appropriateness of the Nutrition Literacy Assessment Tool.

Chin et al. performed a scoping review concentrating on evaluation instruments for general nutrition knowledge among older individuals and their caregivers in various care environments (22). The inquiry was confined to papers published exclusively in the English language. Only methods evaluating general nutrition knowledge were included due to limits in the inclusion criteria. Tools pertaining to other nutritional domains (e.g., nutrition or health literacy, comprehension of food labels, nutritional attitudes, beliefs, and behaviors) and particular subjects (e.g., cardiovascular disease or protein-energy deficiency) were omitted. Obviously, the scope of this study is relatively limited. This paper examines the accessibility of tools, including items and dimensions, along with their reliability and validity, while not extensively addressing their clinical suitability for older adults. Recently, scholars have deepened their research on nutrition literacy assessment tools, and many achievements have emerged. Based on this, timely and re-examination of assessment tools cannot be ignored.

A strong correlation exists between diet, aging, and disease. Nutrition acts as the pivotal connection, significantly influencing the physical health and quality of life of the aged. A thorough and precise evaluation of the nutritional status of the elderly is essential for implementing clinical nutritional interventions and serves as a solid foundation for developing personalized nutritional support regimens. Scoping reviews are an evolving research method whose comprehensive overview of a specific area of inquiry helps to address the shortcomings of current research while also guiding future research directions correctly (23). This study aims to perform a thorough evaluation, carefully gathering and analyzing existing assessment measures for the nutritional literacy of the elderly. It seeks to serve as a benchmark for the advancement of more efficacious evaluation instruments and therapies in the future, and will be utilized in clinical practice to enhance the health and quality of life of individuals.

## 2 Materials and methods

The concepts in Arksey and O′Malley’s research review framework provide guidance for the writing of this review and further refined by using the Preferred Re-porting Items for Systematic reviews and Meta-Analyses extension for Scoping Re-views (PRISMA-ScR) Checklist (23, 24). The protocol for this review was registered with the Open Science Framework: https://osf.io/yv27b (accessed on 25 March 2025).

### 2.1 Identifying the research question

This study poses two research questions: (1) what are the types and characteristics of assessment tools currently applied to nutrition literacy at home and abroad? (2) What are the main assessment components involved within these assessment tools?

### 2.2 Identifying relevant studies

A comprehensive search strategy was developed for seven major databases, including PubMed, Web of Science, the Cochrane Library, Wanfang, China Biology Medicine disc (CBM), China National Knowledge Infrastructure (CNKI) and VIP Database for Chinese Technical Periodicals (VIP). This search encompassed data from the inception of each database up to November 29, 2024, utilizing a blend of controlled vocabulary and keyword searches. The search process adhered to a systematic methodology, with a postgraduate nursing student developing a comprehensive and detailed search strategy. Subsequently, this strategy was reviewed by the team for additional enhancements. A snowballing technique was used for citation tracking, while grey literature was not included in the search process. Finally, the literature was imported into EndNoteX9. English search terms included aged, long-lived, elderly, centenarian, nutritional status, nutrition literacy, nutritional knowledge, food literacy, nutrition, nutrition assessment, nutrition index, mini nutritional assessments, assessment tool, shortlist, assessment instrument and screening. For instance, in PubMed, the search strategy is shown in Table 1.

**Table 1:** Strategy used for searching studies in PubMed.

Process	Search strategy	No
#1	(((“aged”[MeSH]) OR (“elderly”[Ti/Ab])) OR (“long-lived”[Ti/Ab])) OR (“centenarian”[Ti/Ab])	3773154
#2	(((“nutritional status”[MeSH]) OR (“nutrition”[Ti/Ab])) OR (“food literacy”[Ti/Ab])) OR (“nutritional knowledge”[Ti/Ab]))	1420782
#3	(((“nutrition assessment”[MeSH]) OR (“nutrition index”[Ti/Ab])) OR (“mini nutritional assessments”[Ti/Ab])) OR (“nutrition literacy”[Ti/Ab])	20764
#4	(((“screening”[MeSH]) OR (“assessment tool”[Ti/Ab])) OR (“shortlist”[Ti/Ab])) OR (“assessment instrument”[Ti/Ab])	215635
#5	#1 AND #2 AND #3 AND #4	518

### 2.3 Study selection

Inclusion criteria for the literature were as follows: (1) Participants aged 60 years and above, encompassing elderly inpatients, community-dwelling seniors, and individuals residing in nursing homes; (2) Focus on primary literature concerning the development, validation, adaptation, localization, and utilization of the Nutrition literacy Assessment Tool; (3) Study designs encompassed cross-sectional, longitudinal, cohort, and case–control investigations; and (4) Sources included scholarly articles published in academic journals. The exclusion criteria comprised: (1) duplicate publications; (2) lack of access to the full-text article; (3) non-Chinese and English literature; (4) conference abstracts, dissertations, editorials, letters, books and reviews; and (5) comprehensive assessment tools containing nutritional assessment. The screening process involved automated tools to detect duplicate entries. Additionally, two authors independently assessed the titles, abstracts, and full texts of the identified articles, with any discrepancies resolved through consensus among the research team.

### 2.4 Charting the data

All pertinent literature was imported into EndNote X9 for meticulous organization. A structured data extraction form was devised to aid in addressing the research inquiries. Systematic data extraction was carried out on the selected literature, encompassing details such as authors, publication year, country, study design, research objectives, participant demographics, and assessment tool characteristics. Two authors independently extracted the relevant information. Any differences in extracted information were resolved through consensus within the research team, and a comprehensive summary was compiled post-extraction.

### 2.5 Data synthesis

Based on the Consensus-based Standards for the selection of health Measurement Instruments (COSMIN), the assessment instrument was assessed by two authors (Y. X. R. and Y. H. L.). In the event of a disagreement, a third author (K. L.) was consulted to resolve the matter. The initial evaluation of the instrument’s internal structure (structural validity, internal consistency, and cross-cultural validity) was essential for understanding the composition of the items. Internal consistency developed as a crucial measure of the instrument’s quality and reliability. The Cronbach’s coefficient is an essential metric that indicates internal consistency and reliability. The content validity of the assessment instrument is paramount in evaluating the suitability of the tool’s items for the constructs of the target audience and their interests. Ultimately, the residual measurement properties (sensitivity, specificity, and AUC) were also taken into account. Kappa values are predominantly employed to examine the level of agreement among evaluators or the constancy of an evaluator’s assessments of the same object over time. A Kappa value of 0 signifies that the observed consistency corresponds with the anticipated random consistency. A Kappa value of 1 indicates that the observed consistency achieves perfect reliability, devoid of any random.

### 2.6 Summarizing and presenting the results

A descriptive overview of the included studies was conducted, encompassing details on the types of tools, their attributes, core components, and target populations. The results were categorized to enhance the systematic comprehension of the outcomes. An assessment of the prevailing state of existing assessment tools was conducted, with a focus on pinpointing any existing research voids.

## 3 Results

### 3.1 Search results

**Figure 1:**
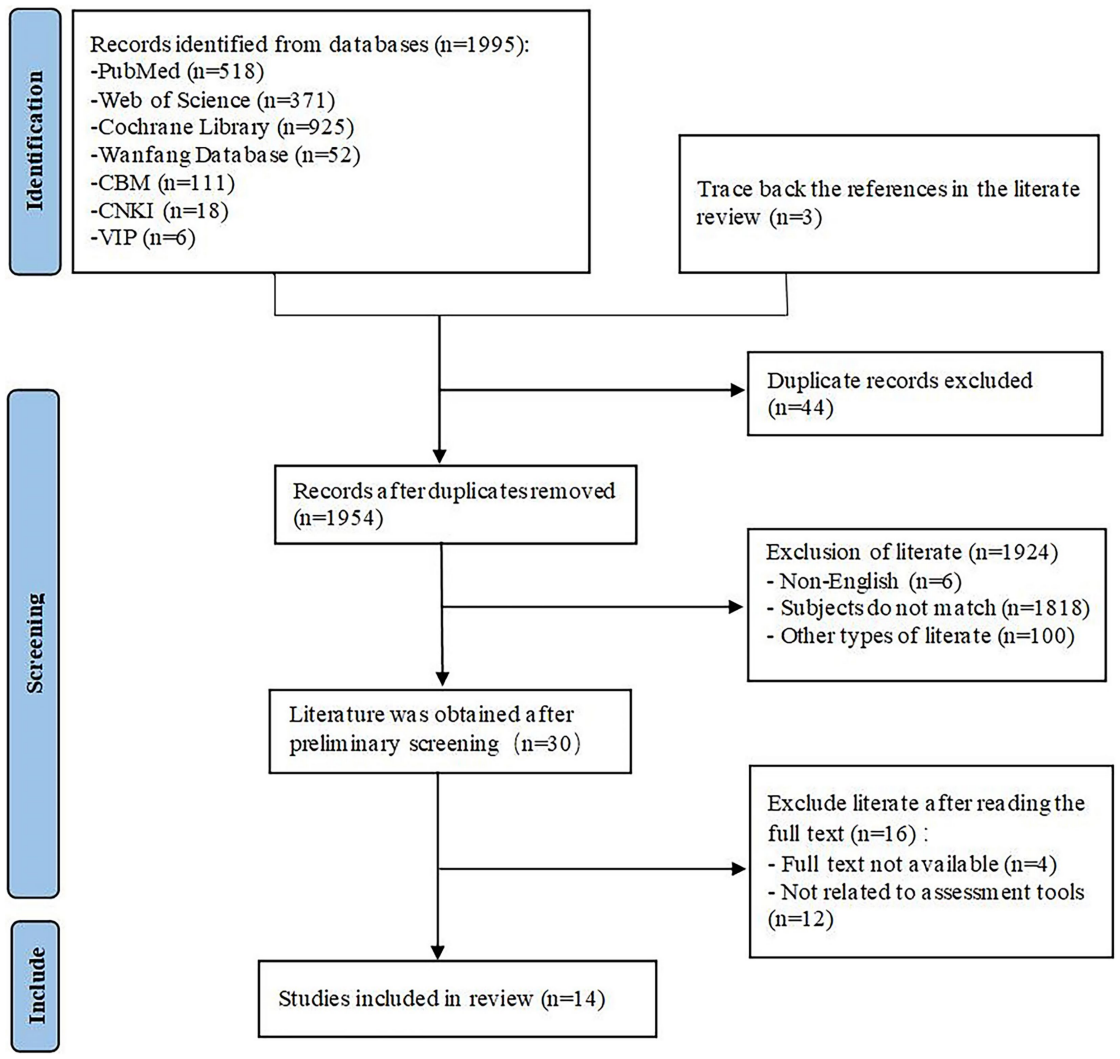
Literature screening process and results.

Following the elimination of duplicates in EndNoteX9, 1954 documents remained for screening. A preliminary evaluation of 1954 papers resulted in the exclusion of items that did not align with the designated topic, specifically those involving non-elderly populations and publications unrelated to nutrition literacy and assessment methods (*n* = 1,818), documents authored in languages other than English (*n* = 6), and additional materials, including reviews and conference abstracts, which did not satisfy the inclusion requirements (*n* = 100). This procedure culminated in the preservation of 30 documents. The 30 retained documents were evaluated by removing those lacking full text or considered unrelated to the assessment instrument. 14 articles were incorporated for the review. The details of the literature screening process and outcomes are depicted in Figure 1.

### 3.2 Basic characteristics of the included studies

A total of 14 articles meeting the requisite criteria were included in the study (18–20, 25–35). Among these, two articles originated from China (29, 33), two from India (27, 32) and two from Nepal (30, 35), with single contributions from Denmark (25), France (26), Japan (18), USA (19), Brazil (28), Iran (20), Singapore (31) and Ethiopia (34). The publication years span from 2003 to 2024, with the predominant study design being cross-sectional studies (18–20, 27–35), inclusive of tool development and validation studies (25, 26). The compilation comprises three articles focusing on tool localization and application (20, 28, 29), four articles on tool development and validation (18, 25, 26, 33), and seven articles on tool application (19, 27, 30–32, 34, 35).

### 3.3 The general characteristics of the nutrition literacy assessment tools

A total of 12 nutrition literacy assessment tools for older adults were included in this study, concentrating on the timeframe spanning 1996–2022. The basic characteristics of these tools are presented in Table 2.

**Table 2:** Chronological overview of the nutrition literacy assessment tool for the elderly.

Author	Year	Tool name	Population	Number of dimensions/entries	Reliability and validity	COSMIN rating	Dimension project	Criteria for judging results	Advantages	Disadvantages
Guigoz (37)	1996	MNA	Elderly people in the community, hospitals and nursing homes	4/18	Cronbach’s alpha 0.7–0.8, sensitivity 96%, specificity 98%, positive predictive value 97%	Very good	(i) Anthropometric indicators; (ii) holistic assessment; (iii) dietary assessment; (iv) self-assessment	Normal (score >24); malnutrition or critical risk (score 17–24); and malnutrition (score <17)	Assessment is comprehensive, multidimensional and clinically applicable. It can be used to treat type 2 diabetes	The evaluation process is complex and time-consuming, some indicators are subjective, special populations are not well targeted, and there is no dynamic evaluation mechanism
Rubenstein (38)	2001	MNA-SF	Elderly people in the community, hospitals and nursing homes	4/6	Cronbach’s alpha 0.7–0.8, sensitivity 98%, specificity 100%, diagnostic accuracy 99%	Very good	(i) Anthropometric indicators; (ii) activity assessment; (iii) eating assessment; (iv) mental-psychological assessment; (v) disease and stress	Scores 0–14 indicate nutritional status: 0 is optimal and 14 poor. Scores 0–7 indicate mild malnutrition; 8–11 are moderate risk; 12–14 good nutritional status	Easy and practical, multi-dimensional assessment, strong standardization	An absence of dynamic assessment capacity, a high degree of subjectivity, an insufficient level of information detail, and poor specificity
Söderhamn (40)	2002	NUFFE	Elderly people in the community, hospitals and nursing homes	4/15	Cronbach’s alpha 0.72; Face validity: over 95% of participants believed the scale reflected their nutritional condition to some extent	Very good	(i) Dietary history; (ii) dietary assessment; (iii) diet-related health problems; (iv) overall assessment	Scores on the scale range from 0 to 30. Higher scores indicate a higher risk of malnutrition. Scores over 13 indicate malnutrition	Well-structured, content-targeted and easy to operate	A suboptimal accuracy, absence of a weighting system, subjectivity in assessment and inadequate dynamic assessment
Kondrup (25)	2002	NRS2002	Elderly people in hospitals	3/3	Cronbach’s alpha 0.70–0.80, AUC 0.934, sensitivity100%, specificity 87%	Very good	(i) Disease severity; (ii) nutritional damage; (iii) age	An integer greater than or equal to three indicates a nutritional risk, while an integer less than three indicates no nutritional risk	Evidence-based medicine foundation, high scientific and reliability, strong operationalization; wide range of application	An inadequate targeting of special populations and limited use of physiological indicators
Bouillanne (26)	2005	GNRI	Elderly people in hospitals	2/4	Cronbach’s alpha 0.70–0.80, AUC 0.830 for discriminating between hospitalization and all-cause death, sensitivity 85.1%, specificity 80%	Very good	(i) Serum protein level; (ii) body weight	As the score decreases, the nutritional risk increases. Scores below 82 indicate high risk, 82–92 indicate moderate risk, 92–98 indicate low risk, and above 98 indicate no risk	Objective, widely used, provides comprehensive disease assessment	The limitations of indicators, the one-sidedness of assessment results, and the lack of harmonized standards
Kruizenga (36)	2005	SNAQ	Elderly people in communities, hospitals and nursing homes in Dutch-speaking areas	−/4	Cronbach’s alpha 0.65–0.85; AUC 0.850 in moderate and severe malnutrition, Kappa 69%	Very good	Appetite	A score of 14 or less indicates a significant nutritional risk, suggesting weight loss within 6 months. A score above 14 indicates a normal appetite and low risk	Focused on appetite assessment, sensitive in predicting nutritional risk and adverse health outcomes, simple and fast, easy to understand and administer	The limited scope, subjective nature, inadequate cultural adaptation, and relatively low predictive power for patients with severe illness or those hospitalized
Diamond (39)	2007	NLS	Elderly people in the community, hospitals and nursing homes	6/28	Cronbach’s alpha 0.84, and S-TOF HLA scores had a Pearson correlation coefficient of 0.61, correlation coefficients ranging from 0.7 to 0.85	Very good	(i) Nutrition information; (ii) healthy eating; (iii) caloric intake; (iv) organic foods; (v) saturated fats; (vi) units of food intake	Total scores range from 2 to 28. Scores of 14 or less indicate insufficient nutrition, while scores of 15–21 critical status, and 22–28 high nutrition	Comprehensive knowledge coverage, targeted questions, good reliability and validity	A single question type, an absence of a difficulty gradient, lack of contextualized design and an absence of cultural sensitivity
Aihara (18)	2011	NLAT	Elderly people in communities, hospitals and nursing homes in Japan	1/10	Cronbach’s alpha 0.86	Absence of validity testing for instruments	Eating habits	The total score ranges from 0 to 10, with 10 representing adequate nutrient nutrition and less than 10 representing limited nutrient nutrition	Focused, with good internal consistency	The presence of robust cultural limitations, lack of dynamic assessment capacity, and an inadequate level of individual variability
Machado (28)	2015	MNA Portuguese Version	Elderly people in communities, hospitals and nursing homes in Portuguese-speaking areas	4/18	AUC 0.832, sensitivity 82.8%, specificity 80.0%	Absence of reliability testing for instruments	(i) Anthropometric indicators; (ii) holistic assessment; (iii) dietary assessment; (iv) self-assessment	24 points or higher indicates malnutrition or critical risk; 17–24 points indicates malnutrition. 17 points or lower indicates malnutrition	Assessment is comprehensive, clear-scoring and clinically useful for type 2 diabetes. It is suitable for Portuguese-speaking countries	Intricate and time-consuming, an insufficient targeting of special populations, and lack of dynamic assessment mechanism
Gao (29)	2015	NUFFE-CHI	Elderly people in communities, hospitals and nursing homes in China	4/15	Cronbach’s alpha 0.65, Split-half reliability 0.67, retest reliability 0.88, CVI 0.83	The tool’s reliability is crucial and demands caution	(i) Dietary history; (ii) dietary assessment; (iii) diet-related health problems; (iv) overall assessment	Scores on the scale range from 0 to 30. Higher scores indicate a higher risk of malnutrition. Scores over 13 indicate malnutrition	Well structured, focused and easy to use	A suboptimal accuracy, absence of a weighting system, subjectivity in assessment and inadequate dynamic assessment
Sharifnia (20)	2019	NLS Persian Version	Elderly people in communities, hospitals and nursing homes in Persian-speaking areas	6/28	Cronbach’s alpha 0.80; Average CVI 0.865	Very good	(i) Nutrition information; (ii) healthy eating; (iii) caloric intake; (iv) organic foods; (v) saturated fats; (vi) units of food intake	Total scores range from 2 to 28. Scores of 14 or less indicate insufficient nutrition, while scores of 15–21 critical status, and 22–28 high nutrition	Comprehensive knowledge coverage, targeted questions, good reliability and validity	A single question type, an absence of a difficulty gradient, lack of contextualized design and lack of cultural sensitivity
Aihemaitijian (33)	2022	NLQ-E	Elderly people in communities, hospitals and nursing homes in China	4/25	Cronbach’s alpha 0.678; RMSEA 0.045, PCFI 0.776, PNFI 0.759	Poor reliability of the tool	(i) Basic demographic information; (ii) knowledge and understanding; (iii) healthy lifestyles and eating behaviors; (iv) skills	The total possible score is 100 points. Higher scores indicate better nutrition literacy	Evaluations are comprehensive, well-structured, relevant, and useful	Limited to a single method, no quantitative indicators, insufficient cultural sensitivity and lack of weighting

-, means none; MNA, Mini-Nutritional Assessment; MNA-SF, Mini Nutritional Assessment-Short Form; NUFFE, Nutritional Risk Screening in the Elderly; NRS2002, Nutritional Risk Screening 2002; GNRI, Geriatric Nutritional Risk Index; SNAQ, Simplified Nutrition Appetite Questionnaire; NLS, Nutrition Literacy Scale; NLAT, nutrition literacy assessment tool; MNA Portuguese Version, Mini-Nutritional Assessment Portuguese Version; NUFFE-CHI, Nutritional Risk Screening in the Elderly-Chinese version; NLS Persian Version, Nutrition Literacy Scale Persian Version; NLQ-E, Nutrition Literacy Questionnaire for Chinese Elderly.

### 3.4 The core elements of the content of the nutrition literacy assessment tools

**Figure 2:**
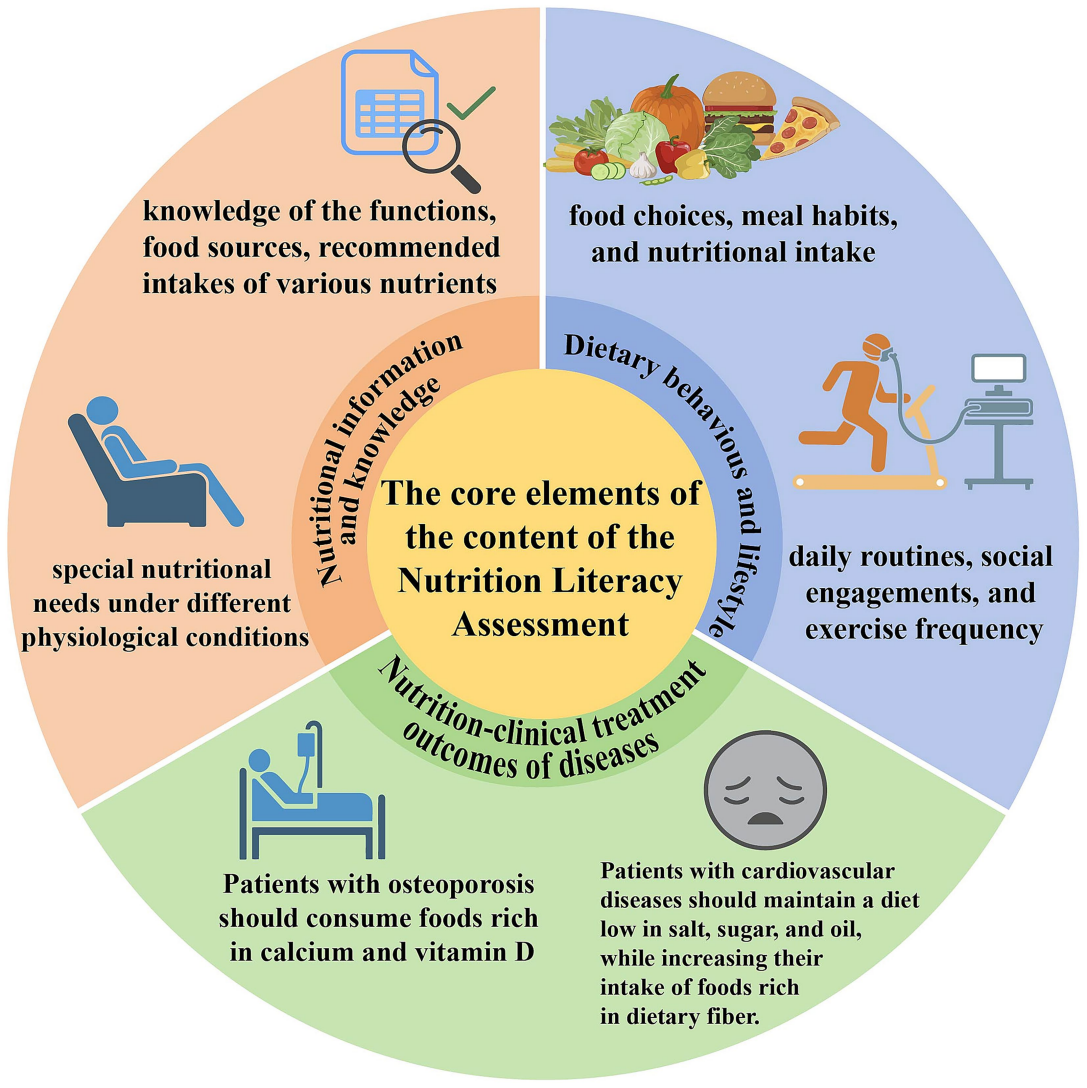
The core elements of the content of the Nutrition Literacy Assessment.

The fundamental components of the Nutrition literacy Assessment Tool for Older Adults consist of three main facets. The primary component includes nutrition information and expertise (18, 20, 28, 29, 33, 36), encompassing the functions, dietary sources, recommended intakes of various nutrients, and specific nutritional requirements under diverse physiological situations. The second component pertains to dietary habits and lifestyles (18, 25, 26, 28, 29, 33, 36–39). Dietary habits are shaped by food, environmental factors, and individual health conditions, encompassing food selections, eating practices, and nutrient consumption. Healthy dietary habits, along with beneficial lifestyles including daily routines, social interactions, and exercise frequency, influence the nutritional depletion, digestion, absorption, and dietary concepts of older adults. Table 2 also displays “nutrition—clinical treatment outcomes of diseases” (20, 26, 28, 29, 33, 37–40). This illustrates the regulatory function of nutrition in the clinical management and treatment outcomes for the elderly population with diseases. In osteoporosis, proactive measures against muscle atrophy and bone degeneration are emphasized, alongside moderate consumption of calcium-rich foods and the nutritional benefits of calcium and vitamin D for maintaining bone density and minimizing fracture risk. For cardiovascular diseases, recommendations include reducing salt, oil, and sugar intake, increasing dietary fiber, and monitoring weight and dietary habits to assist elderly patients in regulating blood pressure and lipid levels. The management of metabolic diseases, such as diabetes, focuses on weight changes, hunger regulation, and sufficient protein intake, while also considering the synergistic effects of diet and medication. Figure 2 clearly illustrates the aforementioned content.

### 3.5 Target population for the nutrition literacy assessment tools

The Nutrition literacy Assessment Tool for Older Adults has demonstrated relevance across three separate demographics. (i) Older adults with specific cultural backgrounds (18, 20, 28, 29, 33, 36): assessment techniques for nutrition literacy that are developed and verified within specific cultural contexts effectively mitigate comprehension bias arising from language barriers or cultural disparities. These methods are better culturally suited and more aligned with the cultural and linguistic backgrounds of the local elderly people. Nonetheless, it is important to recognize that these methods may potentially restrict their application across various cultural contexts. (ii) Elderly individuals within the community (18, 20, 28, 29, 33, 36–40): Assessment tools for older individuals in community settings are practical and viable, covering various characteristics like dietary habits, lifestyle choices, and social interactions. These instruments predominantly manifest as questionnaires, streamlining the evaluation process. Nonetheless, their reach may be constrained, incapable of addressing all cases adequately. (iii) Elderly individuals in hospitals and nursing homes (18, 20, 25, 26, 28, 29, 33, 36–40): owing to illness or physical deterioration, these individuals exhibit limitations in self-care capabilities and consequently necessitate support in daily activities. Therefore, assessment tools must be tailored to their specific requirements, particularly regarding assistance with eating. Moreover, the instruments must be rigorously standardized to enhance healthcare treatments, as discrepancies may arise from human error in evaluations conducted by various individuals. Nevertheless, precisely obtaining body measures in elderly individuals might be challenging due to physical conditions such as disorientation, inability to stand, or edema.

## 4 Discussion

Aging often accompanies the development of chronic conditions, along with prevalent nutritional imbalance, impacting the quality of life in older individuals. Improving nutrition literacy in this population through assessment tools is vital for advancing public health. This review consolidates existing research evidence on nutrition literacy in older adults, presents a comprehensive compilation of assessment tools employed for evaluating nutrition literacy in this demographic, incorporates a total of 12 nutrition literacy assessment tools, and provides a comprehensive analysis of these tools. This review aims to summarize and analyze the essential characteristics of tools utilized for assessing the nutritional literacy of older adults, offering a reference for the future development of more effective assessment instruments and therapies, as well as guiding clinical practice.

Numerous current instruments pertaining to nutrition literacy have insufficient reliability and validity. This study included 12 nutrition literacy assessment methods for older persons, most of which were rigorously evaluated for internal consistency and test–retest reliability. The Cronbach’s alpha of no less than 0.80 indicates sufficient internal consistency of the scale, while an intragroup correlation value of at least 0.75 reflects acceptable retest reliability. The reliability testing excluded the Portuguese version of the MNA. The Cronbach’s alpha for the NUFFE-CHI and the NLQ-E were 0.650 and 0.678, respectively, signifying inadequate internal consistency. The NUFFE-CHI, encompassing risk variables for malnutrition, may be regarded as a causative indicator. Streiner’s criterion indicates that the Cronbach’s coefficient exceeding 0.6 is deemed acceptable for instruments consisting of causal indicators. Consequently, the Cronbach’s coefficient of 0.65 for NUFFE-CHI is considered acceptable (41). The diminished internal consistency of the NLQ-E may be ascribed to variables including a restricted number of questions, overlapping dimensional content, and a limited sample size. This emphasizes the necessity for researchers to improve and optimize the scale’s design to boost its reliability, especially during localization and development. Furthermore, the original study indicated the Cronbach’s alpha of 0.650 for the SNAQ, demonstrating its appropriateness for rapid screening applications. In additional validation experiments, the Cronbach’s alpha of the SNAQ neared 0.850, indicating robust internal consistency. Eleven assessment instruments were subjected to validity evaluations (20, 25, 26, 28, 29, 33, 36–40), primarily utilizing structural and content validity assessments, in addition to correlation analyses with established measurement tools. Nonetheless, discrepancies were evident in the correlation measuring instruments employed for each assessment tool, with variations noted in the substance and markers of their evaluations. The most critical measurement characteristic in a scale is content validity, which has a prominent influence on attributes such as internal consistency and construct validity. COSMIN are selected by consensus based on the same standards, it builds a framework to evaluate measurement tools and their properties, in turn promotes the measurement tool to be more reliable and valid (42). This study selected a total of 12 nutrition literacy assessment tools and found that 8 of them met the COSMIN requirements (20, 25, 26, 36–40). The basis for drawing conclusions includes retesting reliability through description, internal consistency, and rater reliability, and using defined consensus standards to ensure the stability and reliability of the instrument. It can be seen that with the use of these assessment tools, the nutritional status of the elderly can be presented in an accurate, clear and consistent. The consensus standards include a description of the content and prerequisites of the effectiveness criteria, which plays an important positive role in the evaluation of basic information such as nutritional and lifestyle, making it more scientific and accurate. These standards are able to accurately assess the relevant expected materials, while ensuring that the tools can be accepted and normally implemented in the face of differentiated environments Based on this, the effectiveness and practicality of these techniques have been widely recognized.

The assessment of nutrition literacy among the elderly needs to consider factors such as anthropometric measurements, psychological aspects, nutritional knowledge, and dietary patterns from multiple perspectives (43). The development and selection of relevant evaluation tools should also be tailored to the actual situation. Among the 10 multidimensional assessment tools included in this study (20, 25, 26, 28, 29, 33, 37–40), dietary behaviors and nutritional knowledge emerged as the most prevalent dimensions. These two dimensions have become the main focuses of assessing the nutrition literacy of older adults, whereas anthropometric measurements, psychological factors, and self-assessment dimensions were only included by individual assessment tools. Among the single-dimension assessment tools examined (18, 26, 36), the NLAT focuses exclusively on the eating habits of older adults. In contrast, the SNAQ primarily centers on appetite-related factors like appetite level, satiety, food preferences, and food intake quantity, with a narrower scope focused on screening for nutritional risks linked to appetite. However, it should be noted that the aforementioned assessment tools are not designed to evaluate the nutritional status of the elderly in complex life scenarios, potentially leading to biased and general assessment results that may not offer comprehensive guidance for health management. An extensive literature review revealed that the MNA emerges as a comprehensive assessment tool covering diverse factors, including anthropometric measurements, psychological well-being, dietary habits, self-assessment, and nutritional information. This multifaceted approach makes it particularly well-suited to evaluating nutrient literacy in the elderly population. Notably, the MNA evaluation method has already been adopted in the domains of type 2 diabetes (44) and elderly care institutions (45). Furthermore, this study included three localized assessment tools (20, 28, 29). Significantly, the influence of cultural variations on the utilization of the same assessment tool was found to be significant. For instance, the Cronbach’s alpha for NUFFE was determined to be 0.70 (40), however Gao et al. adapted NUFFE for a Chinese context, resulting in the Cronbach’s alpha of 0.65 for NUFFE-CHI (29). A comparison of these two investigations indicates variability in the Cronbach’s alpha of NUFFE, potentially due to a confluence of circumstances. By comparing the two studies, it was found that the Cronbach’s alpha of NUFFE showed variability, which may be the result of multiple factors In the context of different dietary patterns in different countries, cultural dietary differences have an important impact first. For example, Chinese cuisine mainly consists of grains and vegetables, while Swiss cuisine mainly consists of grains, fish, and cheese. Similar differences are common, which poses a serious challenge to using a unified tool for evaluation. Simultaneously, the assessment process of NUFFE involves a subjective assessment component, and the varying cognitive levels of older individuals in different countries, along with their interpretations of nutrition and health, contribute to the variability in NUFFE’s performance. In addition to cultural differences, localization also faces the challenge of adapting the translation of the text and language. Due to variations in grammatical structures and lexical nuances among languages, translation requires a careful approach to avoid semantic divergences that may arise from a literal translation method. For example, translating the SNAQ from Dutch to other languages, with its unique expressions for appetite evaluation, can pose challenges in accurately capturing the original meaning across diverse cultural and linguistic contexts. Furthermore, differences in linguistic habits and cultural backgrounds affect the translation of specialized terminology, potentially creating comprehension hurdles. For this reason, a multidisciplinary translation team including linguists, dietitians and healthcare professionals can be formed to ensure the accuracy and professionalism of the translation. Linguists oversee the linguistic fluency of the translation, while dietitians and healthcare professionals check the translation from a professional perspective to ensure the relevant expressions are accurate. However, the formation of multidisciplinary translation teams presents challenges, such as substantial costs related to validation and the need for enhanced research funding. A strategy to address these challenges involves facilitating resource sharing and minimizing costs through international alliances and the creation of collaborative networks. Upon completing the translation process, backtranslation validation is essential to compare the original tool with the back-translated version and rectify any discrepancies in timing. To tackle the issue of cultural adaptability in assessment tools, it is advisable for international organizations, such as the WHO, to establish guidelines for the cross-cultural validation of nutritional assessment tools for older adults. This should include unifying the report format and standardizing the translation process, culturally adapted lists, and validation outcomes. This will ensure that the assessment tools are equivalent and scientifically valid across different cultures, and that they are more pertinent to older adults regarding linguistic expression and comprehension of content, thereby enhancing their utility and acceptability. This will improve the utility and acceptance of the tool. This approach will more effectively tackle the challenges associated with aging, chronic diseases, and health inequities.

Furthermore, the utilization of AI in the development and optimization of assessment tools has demonstrated notable practicality. In a study by Ali et al., a mobile phone application was developed with the capacity to record and calculate food-related data and generate reports automatically (46). The dietary intake of 54 patients with chronic eye disease was documented for a minimum of 3 days. Subsequently, the recorded data was assessed and compared with results obtained from a traditional paper-based food diary. The results showed good agreement between the paper food diary analyzed by a dietitian and the assessment results calculated by the application. Nevertheless, it is imperative to acknowledge the existing challenges in the realm of AI applications, such as digital age discrimination and insufficient data for the elderly (47). Diverse variables contribute to this issue. Initially, there exists a digital divide among the elderly, characterized by a limited acceptance of AI data collecting tools like smartphones and wearable devices, coupled with a pronounced preference for conventional data collection methods, including follow-up visits by community workers and hospital medical examinations. Moreover, older individuals assign greater importance to privacy and are hesitant to disclose sensitive information like their health, and income, leading to a diminished sample of older participants. In terms of technological investment, technology firms and academic research predominantly focus on younger demographics, emphasizing items like short movies, video games, and other offerings favored by younger users, while overlooking the requirements and preferences of older adults. All sections of society must work together to solve this. First, older people should be more involved in AI development and their data should be used. This technique addresses access, time, training, and finance issues for older persons participating in research and development and collecting more samples (48). Second, creating creative products for older folks with large fonts, great contrast, and a simple voice-operated interface is essential. Age-inclusive design, which prioritizes older persons, must also be promoted. In addition, offering digital literacy training to older persons in rural or isolated regions might mitigate the digital gap.

Despite the availability of several nutrition literacy assessment instruments, deficiencies persist in their applicability, indicator frameworks, and assessment content features. Certain diagnostic tools demonstrate limitations in applicability when utilized with specific subsets of aged adults, including those with cognitive deficits, significant physical fragility, or extended bedridden states. Cognitive decline in the elderly may impair memory, comprehension, and communication, leading to food recall errors and skewed assessment interpretation. This may cause a discrepancy between nutritional status and perception. Bedridden and weak elderly persons may have trouble assessing physical activity indicators like ambulation distance and voluntary intake capacity. The patient’s poor health may impair report accuracy. Assessors should be trained to improve their professional skills and learn geriatric customer-specific communication skills. Simultaneously, multidimensional auxiliary evaluation techniques may be employed alongside objective measurement indications like bioelectrical impedance analysis. This method offers the benefits of non-invasiveness, operational simplicity, and thorough insights into the results (49, 50). Nonetheless, elderly patients with chronic diseases face challenges regarding the applicability of existing assessment tools. Chronic diseases are defined by their prolonged duration and recurrent nature, accompanied by differing nutritional requirements at various stages. Most existing assessment tools are statically designed, making them difficult to adapt. This results in a disconnect between assessment outcomes and clinical nutritional needs, thereby limiting their guiding value (51). Elderly patients with chronic heart failure frequently experience malnutrition and cachexia. Dynamic assessment significantly enhances clinical outcomes. Initiating long-term dynamic monitoring requires an early nutritional assessment to detect nutritional issues. Monitoring a patient’s nutritional status enables the adjustment of intervention strategies to avert deterioration. Classification of patient heart function should inform adjustments in nutritional management and fluid intake. Guidelines from Europe, America, and China recommend restricting fluid intake for patients with severe heart failure to 1.5–2.0 liters daily. Limiting fluid intake is not significant for individuals with mild to moderate heart failure (52–54). Additionally, individualized protein intake management for cachexia symptoms is also necessary. The 2019 HFSA consensus advises that individuals with chronic heart failure maintain a protein intake of no less than 0.8 g·kg^−1^·d^−1^. For individuals experiencing malnutrition or cachexia, a minimum of 1.1 g·kg^−1^·d^−1^ is deemed appropriate (54). While certain researchers have created nutritional assessment scales tailored for heart failure patients, enhancing the precision of nutritional evaluation and offering predictive insights into negative clinical outcomes, limitations persist in the dynamic assessment throughout the progression of the disease (55). COPD is a prevalent respiratory condition characterized by enduring symptoms and restricted airflow. Patients with COPD exhibit a significant risk of malnutrition attributable to metabolic abnormalities and systemic inflammation. Moreover, dietary status significantly influences the progression of illness (56). Therefore, a dynamic system for nutritional assessment is essential. Nutritional support is a critical component of COPD management and should be consistently optimized based on the disease stage and clinical progression. According to evidence-based medical research, stable COPD patients should eat a high-fat diet with limited carbohydrates and more protein. Acute exacerbation patients, except those with hypercapnia, can also benefit from a high-fat, low-carb diet (57). Human energy comes from carbohydrates, fats, and proteins. Carbohydrates have high respiratory entropy, so excessive consumption may increase the ventilatory burden in COPD patients, leading to hypercapnia and respiratory failure. Fats have low respiratory entropy, but excessive intake may cause gastrointestinal discomfort. Insufficient or excessive intake of protein can both lead to the deterioration of the condition. Customized modification is key to nutritional assistance for COPD, as the right ratio of these three components is vital and difficult. The indicator system constitutes the fundamental component of the assessment tool, with weighting serving as its pivotal aspect. Wang et al. emphasize that nutrition literacy is a multifaceted term, including nutritional knowledge, dietary choices, and essential skills, among other factors (58). Its importance in the comprehensive evaluation is inconsistent. An empirical and rational weighting approach can accurately represent each factor’s contribution to nutrition literacy, ensuring that assessment results reflect older adults’ nutrition literacy. In practice, several assessment tools lack a weighting mechanism and possess unclear weight configurations. Due to this shortcoming, assessment results are unreliable and specific intervention methods for important weak links are difficult to design, resulting in ineffective interventions. By engaging domain experts, the Delphi technique improves nutrition literacy evaluation tools. This ensures weight legality and justification (59). The dimensional components of the tools indicate that the current evaluation instruments are predominantly generalizable, emphasizing basic nutritional knowledge, overall nutritional status, and dietary behaviors. Moreover, for geriatric patients with chronic illnesses, the lack of signs for managing nutritional complications in existing assessment systems is a significant shortcoming in clinical nutrition evaluation. Patients with geriatric chronic illnesses experience prolonged disease trajectories and intricate health conditions, rendering them susceptible. Research indicates that dietary supplementation is a crucial method for mitigating difficulties associated with chronic diseases (60). Diabetic foot ulcers represent significant difficulties for individuals with diabetes, and a proper diet is especially crucial for these people. A survey indicated that diabetic foot patients have a daily protein intake of 104 ± 49 g, which falls short of the optimal range for effective wound healing. The energy contribution ratio was 19%, aligning with the recommended range for the general population (61). Consequently, folic acid is deemed crucial for tissue repair, and inadequate consumption may negatively impact wound healing; similarly, nutritional deficiency is significantly associated with the onset and progression of chronic heart failure, and adequate nutritional support can enhance the prognosis of critically ill patients suffering from heart failure. Individuals with heart failure and cachexia typically demonstrate inflammatory responses and metabolic abnormalities. The compromised state of the myocardium may be enhanced through the supplementation of nutrients, including selenium, vitamins, and antioxidants (62). The Mediterranean diet (MedDiet) and the Dietary Approaches to Stop Hypertension (DASH) are the most extensively researched dietary strategies for the prevention of heart failure and enhancement of prognosis. Both diets advocate for an increased consumption of fruits, vegetables, whole grains, and legumes, alongside a reduction in saturated fat intake. The implementation of these two diets has the potential to enhance the quality of life in elderly patients, decrease the incidence of heart failure, and reduce hospitalization rates (63).

The evaluated content’s characteristics reveal that current assessment tools primarily emphasize fundamental nutrition knowledge (including food sources and nutrient functions) and basic behavioral descriptions (such as food choices and eating frequencies), while neglecting the assessment of the elderly’s capacity for critical evaluation of nutrition information. Numerous unsubstantiated claims regarding food and nutrition are disseminated. Oral misperceptions have evolved into “fallacies,” which are deeply rooted in the senior demographic and affect their dietary decisions (64–66). In the digital era, individuals with unethical intentions exploit platforms to mislead older adults into purchasing costly health products (67). Inadequate nutritional literacy hinders the elderly’s ability to identify misinformation, resulting in financial losses and potential neglect of routine treatments as they depend on counterfeit products, thereby jeopardizing their health and elevating the risk of requiring clinical intervention. Consequently, accurately interpreting nutritional information is crucial for the effective implementation of clinical nutritional interventions. This ability is essential for the effective application of nutritionists’ recommendations in daily life. Current nutrition literacy assessment tools predominantly overlook the evaluation of this capability, especially with chronic disease-specific nutritional knowledge, exposing a significant disparity with clinical nutrition practice. Carbohydrates serve as the primary energy source for the human body, and their intake directly influences post-meal blood sugar levels. However, different carbs have varied effects on different people (68–71). Personalized dietary plans should prioritize carbohydrate sources that are high in dietary fiber, vitamins, and minerals, while minimizing added sugars, fats, and sodium. Additionally, avoid sugar, fat, and sodium-rich diets (68–70, 72, 73). People with diabetes should consume 130 grams of carbs daily, 40–50% of total energy (74). Elderly diabetics must also plan their carbohydrate intake. The exam focuses on “controlling sugar intake,” ignoring clinical nutrition expertise. This gap underscores the disconnect between assessment tools and the requirements of clinical practice. Acute exacerbations of COPD are frequently triggered by respiratory viral or bacterial infections, leading to heightened airway inflammation (75). Vitamin D metabolites enhance antiviral and antimicrobial responses while mitigating inflammation (76). Research indicates that vitamin D supplementation can effectively lower the risk of moderate to severe acute COPD exacerbations in patients with serum 25-hydroxyvitamin D levels below 25 nmol/L, while showing no effect in those with higher levels (77). Incorporation of vitamin D into the diet and monitoring of serum 25-hydroxyvitamin D levels are essential components of clinical nutritional treatment for patients with COPD. Maintaining adequate vitamin D levels through dietary intake or clinical nutritional supplements may prevent acute exacerbations of COPD and enhance quality of life.

The existing tools for evaluating the nutrition literacy of the elderly exhibit considerable limitations regarding their applicability, indicator systems, and the characteristics of assessment content. This directly impacts the accurate evaluation of the nutritional status of the elderly and further limits the execution of clinical nutrition initiatives. Consequently, the development or optimization of pertinent assessment tools is essential. Ensuring the precise alignment of tools with clinical nutrition requirements can enhance the accuracy of nutritional management for the elderly, thereby improving the guidance and effectiveness of clinical nutrition practice.

## 5 Strengths and limitations

This study follows the guidelines outlined by Arksey et al. (24) for conducting and reporting scoping reviews, focusing on summarizing and analyzing the included literature without assessing its quality. This study fills a gap in the current research by providing the first systematic analysis of how a nutritional assessment tool was adapted to different cultural contexts. It also highlights shortcomings in diabetes testing.

However, this scoping review has limitations. It is important to note that the predominant focus of the included tools is on cross-sectional studies, thereby lacking longitudinal data. Secondly, the literature was solely obtained from English and Chinese databases, potentially leading to the omission of significant cross-cultural assessment instruments. This constraint is exacerbated by the possible existence of linguistic bias in the research. Additionally, the exclusion of gray literature may have resulted in an underestimation of publication bias.

## 6 Conclusion

This study comprehensively evaluated the assessment instruments for nutrition literacy in the elderly, emphasizing their limitations regarding application, index system, and characteristics of assessment material in senior patients with chronic conditions. It underscored the necessity for exact alignment with clinical nutritional requirements. The nutrition literacy evaluation techniques for the elderly are currently aligned with clinical requirements and are undergoing continual innovation, indicating a positive developmental trajectory. In the future, interdisciplinary resources must be amalgamated to create and enhance accurate and pragmatic assessment tools for clinical nutritional risk classification and the development of individualized intervention strategies. The advancement of evaluation tools can be facilitated by integrating artificial intelligence technologies, hence augmenting their utility in clinical nutritional practice.
